# The actin binding protein drebrin helps to protect against the development of seizure-like events in the entorhinal cortex

**DOI:** 10.1038/s41598-021-87967-5

**Published:** 2021-04-21

**Authors:** Alexander Klemz, Patricia Kreis, Britta J. Eickholt, Zoltan Gerevich

**Affiliations:** 1 Institute of Neurophysiology, Charité—Universitätsmedizin Berlin, Charitéplatz 1, 10117 Berlin, Germany; 2 Institute of Biochemistry, Charité—Universitätsmedizin Berlin, 10117 Berlin, Germany

**Keywords:** Actin, Biochemistry, Proteins, Cell biology, Cytoskeleton, Mechanisms of disease, Neuroscience, Spine regulation and structure, Physiology, Neurophysiology, Diseases, Neurological disorders, Epilepsy

## Abstract

The actin binding protein drebrin plays a key role in dendritic spine formation and synaptic plasticity. Decreased drebrin protein levels have been observed in temporal lobe epilepsy, suggesting the involvement of drebrin in the disease. Here we investigated the effect of drebrin knockout on physiological and pathophysiological neuronal network activities in mice by inducing gamma oscillations, involved in higher cognitive functions, and by analyzing pathophysiological epileptiform activity. We found that loss of drebrin increased the emergence of spontaneous gamma oscillations suggesting an increase in neuronal excitability when drebrin is absent. Further analysis showed that although the kainate-induced hippocampal gamma oscillations were unchanged in drebrin deficient mice, seizure like events measured in the entorhinal cortex appeared earlier and more frequently. The results suggest that while drebrin is not essential for normal physiological network activity, it helps to protect against the formation of seizure like activities during pathological conditions. The data indicate that targeting drebrin function could potentially be a preventive or therapeutic strategy for epilepsy treatment.

## Introduction

Epilepsy is a disease of high prevalence (~ 1%) with a third of the patients having pharmacoresistant seizures intractable to available treatments^1^. In addition, people with epilepsy often have long-term cognitive impairments such as memory loss, learning disabilities and behavioral disorders frequently correlating with the frequency and severity of epilepsy^2^. Current antiepileptic drugs symptomatically suppress the seizures without affecting the underlying mechanisms of epileptogenesis and brain injury. Comorbidities of epilepsy, such as cognitive impairments, are also rarely targeted by antiepileptic strategies, although they can be as disabling as the seizures themselves^1^.

Dendritic abnormalities have been increasingly observed in both epilepsy patients and animal models^3^. After seizures, a transient beading of the dendrites occurs followed by a more persistent loss of dendritic spines^4,5^. This dendritic spine loss is well documented also in neocortex distant from the epileptic focus^6^ but it is still not clear what is the role of these dendritic abnormalities in promoting epileptogenesis. One possibility is that spine loss is epileptogenetic and enhances the probability of future seizures by disturbing the fine-tuned balance between excitatory and inhibitory circuits, especially when inhibitory inputs are more affected. However, it is also possible that a loss of spines and synapses is a consequence of the seizures with a beneficial role in suppressing seizures, by inhibiting synaptic transmission and the propagation of seizures in the brain.

The cytoskeletal protein actin, existing in depolymerized monomeric (G-actin) and stable filamentous form (F-actin), plays a major role in generating the structural support for dendrites and spines. The organization and turnover of actin filaments within dendritic spines is modulated by actin-binding proteins, such as the developmentally regulated brain protein (drebrin)^7^. In its function as actin filament modulator, drebrin stabilizes actin filaments and inhibits their depolymerisation in spines^7^.

Several studies have reported changes in synaptic transmission and plasticity following drebrin down regulation or drebrin loss. Downregulation of drebrin reduces dendritic spine density, alters spine morphogenesis and inhibits both glutamatergic and GABAergic synaptic transmission in cultured hippocampal neurons^8–10^. Drebrin knock out mice as well as depletion of a splice variant of drebrin in mice has been shown to cause LTP impairment combined with decreased spine density^11,12^. Additionally, depletion of one of the splice variants of drebrin show impaired context dependent fear conditioning^12^. We recently generated a drebrin deficient mouse line and observed no changes in spine morphogenesis or glutamatergic transmission in young adults, suggesting that loss of drebrin alone is not sufficient to induce glutamatergic synaptic dysfunction^13^. We surmised that a specific drebrin KO phenotype only becomes evident under certain pathophysiological conditions where the loss of drebrin cannot be compensated.

Decreased levels of drebrin in the brain were shown in states with high epilepsy prevalence^14^ such as Alzheimer’s disease^15^ and Down's syndrome^16^ and a lower drebrin level in the hippocampi of temporal lobe epilepsy patients was associated with more frequent seizures^17^. Decrease and reactivation of drebrin expression in post-status epilepsy models have been described already^18–20^, but it still remains unclear whether the changes are pro- or antiepileptogenic and if they are the consequence of the seizures or play a role in the epileptogenesis.

Here we measured the effect of drebrin ablation on increased neuronal network activity in physiological and pathological conditions. First, we induced gamma oscillations in hippocampal slices from wild type (WT) and drebrin knockout (KO) mice. Gamma oscillations represent physiological synchronization of neuronal activity at frequencies between 30 and 90 Hz and are associated with higher cognitive tasks such as sensory processing, working memory, attention, learning and memory^21,22^. Second, we induced epileptiform activity in the entorhinal cortex in vitro^23,24^ and analyzed whether the loss of drebrin affects the emergence of epileptiform discharges. We found that drebrin ablation was largely compensated in neural networks when activity remained in a physiological frame; however, networks without drebrin developed seizure-like events with shorter onset latency and higher incidence.

## Results

### Drebrin loss does not alter gamma oscillations in hippocampal slices

**Figure 1:**
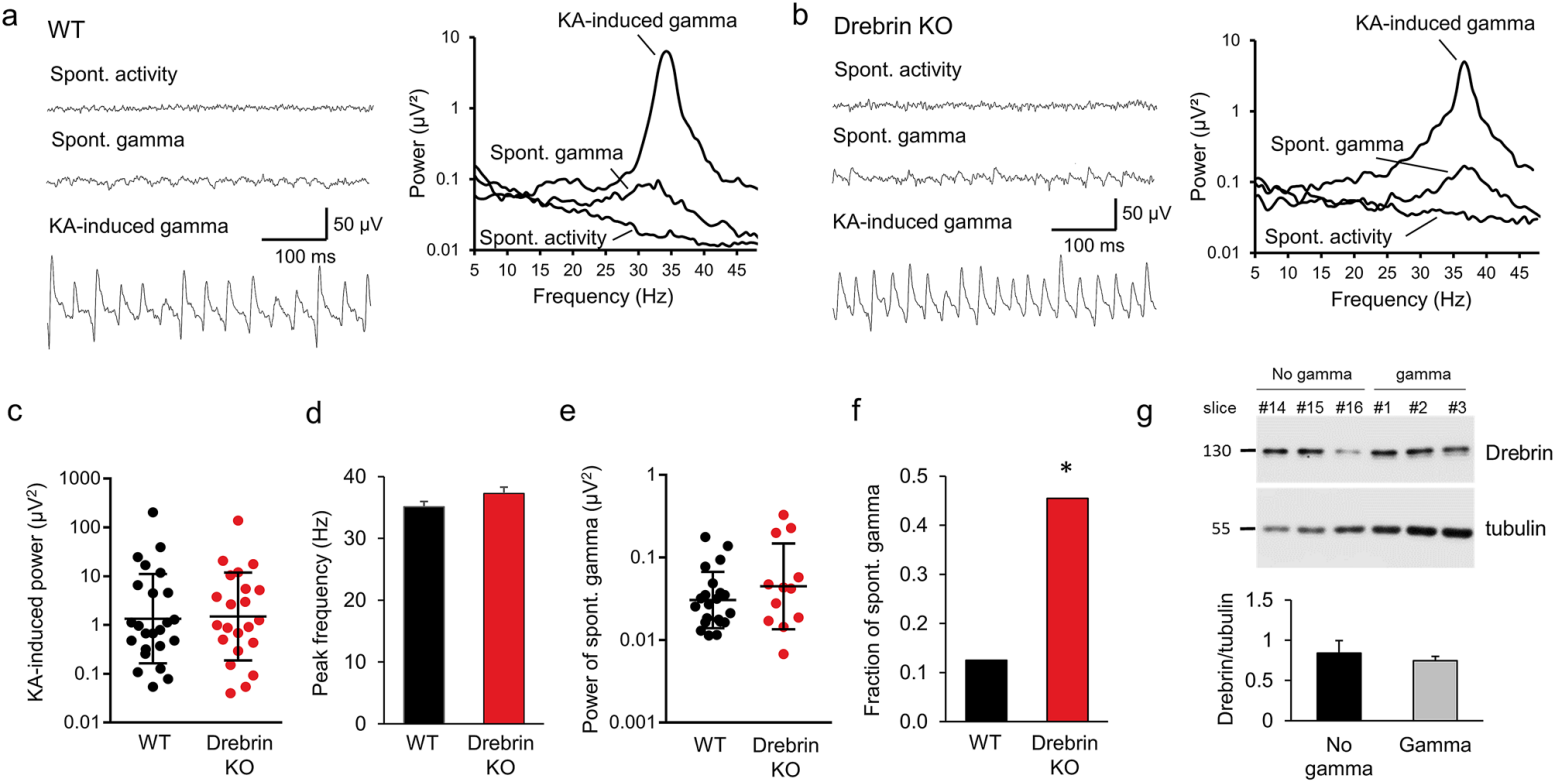
Effect of drebrin loss on gamma oscillations in the CA3 area of the hippocampus. (**a**,**b**) Original traces (left) and power spectra (right) before and after induction of gamma oscillations by kainate (KA; 500 nM) in slices from wildtype (WT; **a**) and drebrin knockout (KO; **b**) mice. Traces were lowpass filtered at 200 Hz. In a fraction of the slices, spontaneous gamma oscillations emerged before the application of KA. (**c**) Absolute power of induced gamma oscillations in WT (black) and drebrin KO (red) mice. (**d**) Peak frequency of induced gamma oscillations in WT (black) and drebrin KO (red) mice. WT: 35.1 ± 0.9 Hz, n = 24; KO: 37.3 ± 1.1 Hz, n = 22; p = 0.11. (**e**) Power of spontaneous gamma oscillations in WT (black) and drebrin KO (red) mice. Geometric mean of power in WT: 0.030 µV^2^, SD factor: 2.19, n = 21; KO: 0.045 µV^2^, SD factor: 3.3, n = 12, p = 0.27. (**f**) Fraction of slices generating spontaneous oscillations in WT and drebrin KO. WT: 3/24 (12.5%), KO: 10/22 (45.5%), p = 0.021. (**g**) Drebrin expression in hippocampal slices responding or not to KA-induced gamma oscillations. Hippocampal slices, previously stimulated with KA to develop gamma oscillations, were probed for drebrin proteins. We compared responder (gamma; grey) to non-responder (no gamma; black) slices. All samples were run on the same gel and transferred on the same blot. Data shown are representative of 17 slices from 4 experiments. Western blot band densitometry revealed no altered drebrin levels in kainate induced gamma oscillations tissue compared to no gamma oscillations (gamma: drebrin/tubulin: 0.75 ± 0.05, n = 17; no gamma: drebrin/tubulin: 0.84 ± 0.15, n = 6; p = 0.59). Bars show mean ± SEM. * p < 0.05. Full-length blots are presented in Supplementary Fig. 1.

Occurring in different regions of the brain, gamma oscillations are physiological rhythmic fluctuations of field potentials enabling the temporal synchronization of neuronal activity within and across groups of neurons. In the cortex and hippocampus, they are generated by recurrent rhythmic synaptic connections between pyramidal cells and perisomatic parvalbumin-containing basket cells^25^. Drebrin is expressed in the dendrites of both glutamatergic and GABAergic neurons at the site of excitatory synapses^26^. Given that drebrin is a regulator of synaptic transmission and that changes in synaptic morphology are associated with memory consolidation, we tested if loss of drebrin influences the development of gamma oscillations in the CA3 region of the hippocampus, where gamma oscillations are primarily generated and have highest amplitudes^27^. We induced gamma oscillations by bath application of kainate (KA, 500 nM) onto hippocampal slices from WT and KO mice (Fig. 1). The responder rate of the slices was comparable in WT and KO animals (WT: 75.0%; KO: 68.8%; p = 0.782, not shown). The induced oscillations had similar peak power from WT (geometric mean: 1.35 µV^2^, SD factor: 8.21, n = 24) and KO animals (1.50 µV^2^, SD factor: 7.96, n = 22) without statistical difference between the two groups (p = 0.76; Fig. 1c). Similarly, there was no difference in peak frequency (Fig. 1d), Q factor (WT: 8.19 ± 1.21; KO 8.57 ± 1.44; p = 0.84; not shown) and half bandwidth of the oscillations (WT: 8.6 ± 2.1 Hz; KO: 6.8 ± 1.0 Hz; p = 0.45; not shown).

Similar to the induced gamma power, we observed no differences in spontaneous network activity measured prior to the induction of oscillations with KA in WT and KO mice (Fig. 1a,b,e). In a fraction of slices, spontaneous oscillations were recorded before the application of KA. Similar to the induced gamma oscillation, the peak power of these spontaneous oscillations did not differ between WT and KO (geometric mean WT: 0.08 µV^2^, SD factor: 1.75, n = 3; KO: 0.126 µV^2^, SD factor: 4.09, n = 10; p = 0.63, not shown). Likewise, we did not find any differences in the peak frequency, half bandwidth and Q factor of the spontaneous oscillations between WT and KO (frequency WT: 29.7 ± 1.3; KO: 33.1 ± 1.4; p = 0.24; half bandwidth WT: 19.2 ± 8.8; KO: 15.3 ± 3.0, p = 0.59; Q factor WT: 3.0 ± 1.8; KO: 3.2 ± 0.8, p = 0.91, not shown). Interestingly, however, the fraction of slices showing spontaneous gamma oscillations in drebrin KO mice were significantly higher suggesting an increase in neuronal excitability when drebrin is absent (Fig. 1f).

In summary, our results indicate that drebrin loss does not influence the amplitude of gamma oscillations in the hippocampus, however, increased the likelihood of spontaneous oscillation generation in brain slices.

### Gamma oscillations do not alter drebrin protein levels in hippocampal slices

We previously demonstrated that regulation of drebrin protein stability is linked to increased neuronal activity and may protect from synaptic dysfunction^28,29^. We therefore investigated whether the emergence of gamma oscillations altered the protein levels of drebrin in hippocampal slices from WT mice. Quantitative assessments by western blotting showed no difference in the amount of drebrin protein following development of gamma oscillations compared to slices where no oscillations were induced. These results indicate that permanent gamma oscillations lasting for more than three hours did not influence drebrin protein expression in the hippocampus (Fig. 1g).

### Drebrin loss favors the development of epileptiform activity in the medial entorhinal cortex

**Figure 2:**
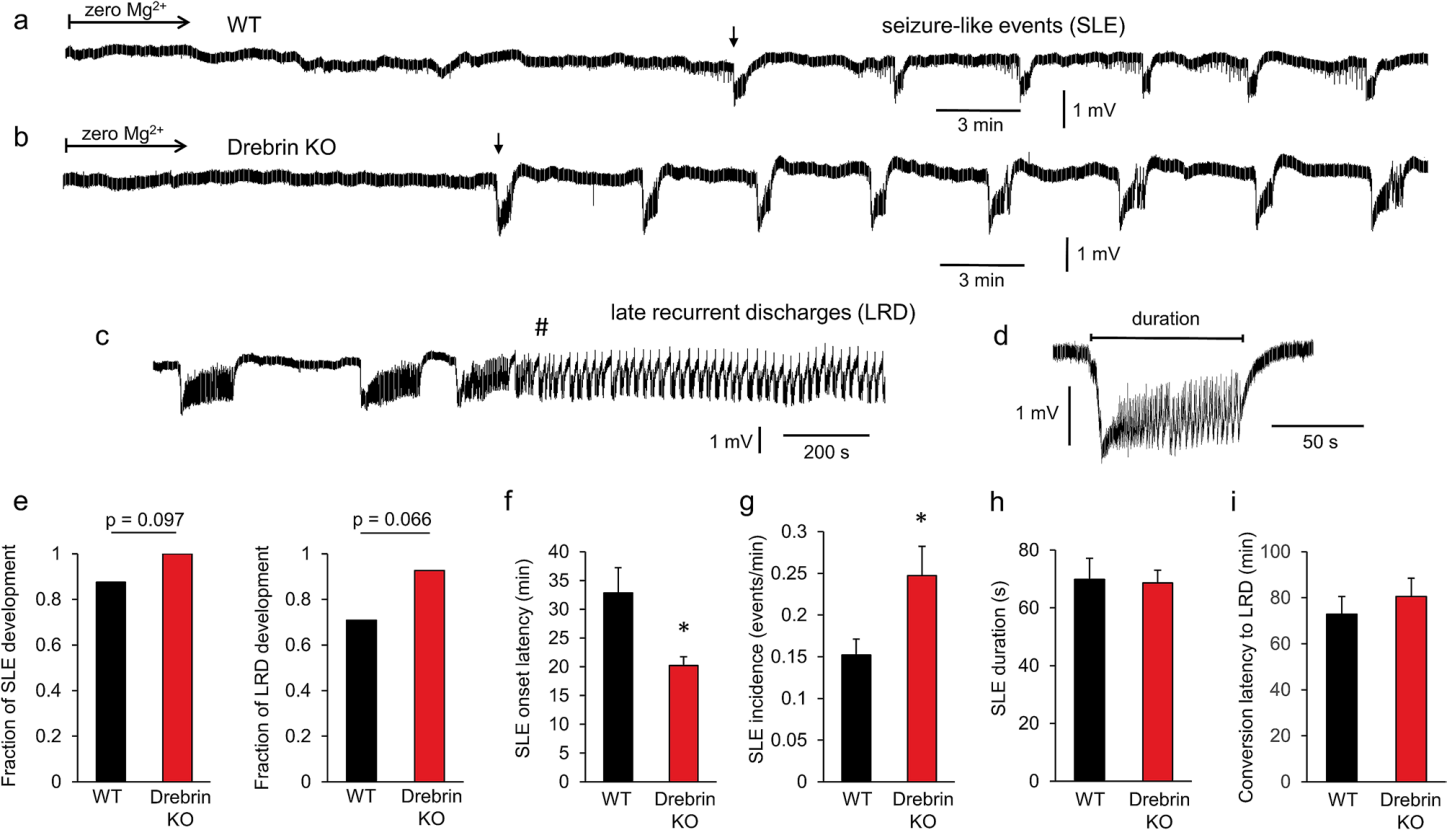
Epileptiform activity of WT and drebrin KO mice in medial entorhinal cortex slices induced by omission of Mg^2+^ from the bath solution. (**a**,**b**) Original field potential traces of seizure-like events (SLEs) characterized by negative potential shifts superimposed by high-frequency oscillations developed after the onset latency (first SLEs marked with an arrow) in WT (**a**) and drebrin KO (**b**) animals. (**c**) Ongoing omission of Mg^2+^ from the bath resulted in conversion of the SLEs into continuous late recurrent discharges (LRDs) after the LRD onset latency (marked with #). (**d**) Duration of one SLE. (**e**) Proportion of slices developing seizure-like-events (SLE; left; WT: 87.5% (21 out of 24) of the slices, KO: 100% (27 out of 27) of the slices; p = 0.10 compared to WT) and continuous late recurrent discharges (LRD; right; WT: 70.8% (17 out of 24), KO: 92.6% (25 out of 27); p = 0.07 compared to WT) after removing Mg^2+^ from the bath solution (**f**) Drebrin KO shortened the SLE onset latency (WT (black): 32.8 ± 4.4 min, n = 21; KO (red): 20.2 ± 1.5 min, n = 27; p = 0.01). (**g**) Drebrin KO increased SLE incidence. WT: 0.152 ± 0.019 min^−1^, n = 21, KO: 0.247 ± 0.035 min^−1^, n = 26, p = 0.03. (**h**) Drebrin KO did not change the duration of single SLEs (WT: 69.9 ± 7.3 s, n = 21, KO: 68.6 ± 4.4 s, n = 27; p = 0.88 compared to WT). (**i**) Drebrin KO did not affect the LRD onset latency (WT: 72.8 ± 7.7 min, n = 17, KO: 80.5 ± 8.0 min, n = 25, p = 0.51 compared to WT). Bar graphs show mean ± SEM. *p < 0.05.

While drebrin KO did not affect the physiological gamma oscillations, we observed more frequent development of spontaneous oscillations before the pharmacological induction suggesting an increased excitability in hippocampal slices. In following experiments, therefore, we investigated whether the loss of drebrin affects the development of pathophysiological epileptiform activity in the medial entorhinal cortex. This area is characterized by generation of seizure-like events (SLEs) with the lowest threshold within the temporal lobe^30–32^. We induced seizure-like activity by omitting Mg^2+^ from the ACSF and observed that SLEs appeared within tens of minutes after induction. SLEs further converted into continuous late recurrent discharges (LRD) with continued omission of Mg^2+^ from the bath solution (Fig. 2).

Analyzing the fraction of slices developing SLEs and LRDs in all investigated entorhinal cortex slices revealed that SLEs and LRDs appeared more often in KO compared to WT animals, although the difference was not significant (SLE: p = 0.09, LRD: p = 0.07 compared to WT; Fig. 2e). However, the onset of SLEs was significantly shorter (Fig. 2a,b,f) and SLEs were more frequent in KO slices compared to WT (Fig. 2g). SLE duration did not differ between WT and drebrin KO slices (Fig. 2h). In contrast to the SLEs, development of LRDs (conversion latency of SLE into LRD) was not different between WT and KO animals (Fig. 2i). These results suggest that while physiological network activity was not affected by drebrin loss, seizure-like activity appeared earlier and more often in drebrin KO mice suggesting that the neural networks without drebrin are more susceptible to develop pathophysiological epileptiform activity.

### Epileptiform activity does not alter drebrin protein levels in medial entorhinal cortex slices

**Figure 3:**
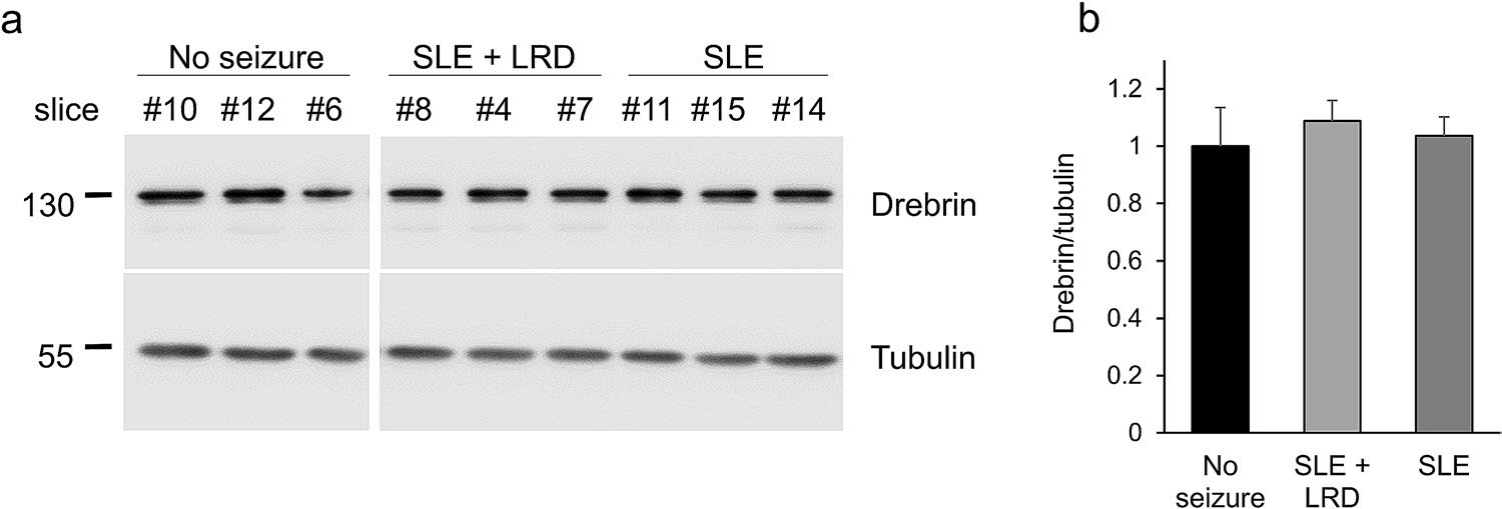
Drebrin expression following epileptiform activity in the medial entorhinal cortex. (**a**) Representative western blot shows levels of drebrin and tubulin in slices that previously developed seizure like events (SLEs) with or without late recurrent discharges (LRDs). All samples were run on the same gel and transferred on the same blot. Separation with a white line indicates cropping of the same blot. (**b**) Quantification of the drebrin/tubulin levels shows no effect of seizure-like activities on drebrin expression (drebrin/tubulin: no seizure (black): 1.0 ± 0.13, n = 6, SLE + LRD (light grey): 1.09 ± 0.07, n = 16, p = 0.54, only SLE (dark grey): 1.04 ± 0.07, n = 4, p = 0.84). Full-length blots are presented in Supplementary Fig. 2.

Previous studies describe decrease in drebrin protein levels in response to KA- or pilocarpin induced seizures^18–20^. We thus set out to investigate whether the emergence of SLEs and LRDs has an impact on drebrin protein levels in the entorhinal cortex of WT mice. SLEs or LRDs did not alter drebrin levels compared to slices without epileptiform activity during the time window of over three hours of recordings (Fig. 3).

## Discussion

We have investigated the effects of drebrin loss on physiological and pathophysiological network activities in hippocampal and entorhinal cortex slices, respectively. While drebrin KO did not alter hippocampal gamma oscillations, it enhanced the susceptibility of entorhinal cortex slices to develop SLEs. In drebrin KO mice, SLEs developed faster and with higher incidence compared to WT mice. Although previous studies reported a decrease in drebrin protein and mRNA levels in epilepsy pathologies^18–20^, we did not find alteration of drebrin expression following in vitro physiological or pathophysiological network activities. The results suggest that drebrin could play a role in controlling the excitation in neuronal circuits and the development of pathological network activity. The results support previous studies showing that drebrin deficiency alone is not sufficient in causing synaptic dysfunction in physiological conditions^13^ and may require additional burdens such as increased excitability to observe a drebrin deficient phenotype.

Our findings demonstrate that gamma oscillations do not differ in drebrin KO animals from those in WT mice. In addition, the induction of gamma oscillations in WT animals did not affect drebrin protein amount in the hippocampal slice, suggesting that drebrin neither modifies gamma oscillations nor gets modified by the evoked oscillatory activity. Although drebrin is expressed in both excitatory and inhibitory neurons^26^ and drebrin downregulation inhibits both glutamatergic and GABAergic synaptic transmission^8–10^ our data suggest that loss of drebrin does not affect normal physiological neuronal activity such as gamma oscillations. The lack of change observed for gamma oscillations in drebrin deficient mice may be due to the presence of compensatory mechanisms by other actin binding proteins enabling activation of alternative pathways to safeguard actin cytoskeleton dynamics. In this case an abnormal phenotype may only be observed in conditions when drebrin cannot be compensated for such as disease or stress.

In these lines, our results further indicate that drebrin loss augmented the susceptibility of neurons to develop seizure-like activity. The results suggest that neuronal networks with no drebrin expression are more likely to develop SLEs. Pathological network events such as hypersynchronized epileptiform activity also depend on GABAergic interneurons. One function of interneurons under normal conditions is to restrain seizure activity by feed-forward inhibition^33^ and failure of this restraint can lead to faster spread of seizures in the brain. GABAergic interneurons are very diverse and at present, no data are available on the expression of drebrin in different interneuron cell types in the brain. However, the loss of drebrin may alter the development of neuronal circuits leading to an imbalance between excitation and inhibition, increased excitability and easier seizure evolution. This is corroborated by our finding that drebrin loss increases the probability of the CA3 network to generate gamma oscillations without exogenous induction. Interestingly, in a recent publication, authors detected anti-drebrin autoantibodies in patients with adult onset epilepsy and suspected encephalitis. Exposure of hippocampal neurons to anti-drebrin autoantibodies resulted in aberrant drebrin distribution within neurons and network hyperexcitability^34^. These results are in accordance with our results and suggest that drebrin dysfunction can lead to impaired synaptic connectivity and increased seizure activity.

There is increasing evidence for dendritic spine abnormalities in the epileptic neocortex and hippocampus, including changes in both structure and number of dendritic spines. Dendritic spine abnormalities were observed in neurodegenerative diseases that have an increased risk of seizures such as Alzheimer’s disease^35^ and juvenile Huntington’s disease^36^. Genetic disorders with dendritic spine abnormalities were also documented to have a high epilepsy prevalence^14^. Among them, Fragile X syndrome, Rett syndrome, tuberous sclerosis and Down syndrome are all characterized by altered spine morphology and, with the exception of Fragile X syndrome, a decreased spine density^16,37–39^. Spine loss and swelling of dendrites have been frequently observed in neocortical and hippocampal pyramidal cells in patients with temporal lobe epilepsy^4,6^. Morphological and structural changes of spines are tightly coupled to reorganization of the actin cytoskeleton mediated by actin-binding proteins^40–42^. In accordance with this, epilepsy is associated with detectable changes in the expression of different actin-binding or their upstream proteins. KA-induced seizures have been shown to activate the actin-depolymerizing protein cofilin and cause a loss of stable actin filaments^43^. Human temporal lobe epilepsy is also associated with decreased expression of reelin, a cofilin phosphorylating and inactivating protein in the hippocampus^44^. Profilin, a protein essential for actin polymerization was also found to be reduced in the hippocampus of temporal lobe epilepsy patients^45^. These findings suggest that epilepsy patients have less stable actin filaments within their spines. In line with this, a recent study found that lower drebrin levels in the hippocampi of temporal lobe epilepsy patients were associated with higher seizure frequency and less neuron survival^17^. These dendritic changes may represent a trait in the pathophysiology of seizure development or a consequence of them or even a compensatory response as a form of homeostatic plasticity to dampen excessive neuronal excitability^14^. Our data on drebrin KO mice suggest that less stable actin filaments in the spines increase the excitability and the probability of seizure-like activity. On the contrary, we did not observe alterations in drebrin expression in the entorhinal cortex after three hours of seizure-like activity, however, we investigated drebrin levels in the whole slice and cannot exclude local or subcellular alterations of drebrin levels after seizures in the hippocampal formation. Previous studies on animal models reported decreased drebrin expression in the hippocampus two hours after systemic KA- or pilocarpin induced seizures^18–20^, possibly by ERK-mediated phosphorylation and activation of the calcium-dependent phosphatase calpain-2^20^. The initial reduction was followed by the recovery of drebrin levels in the chronic phase of pilocarpine-induced seizures suggesting the crucial role of drebrin in reactive synaptic plasticity^18,19^. Another recent study, showed a negative correlation between drebrin expression and seizure frequency in particular in the dentate gyrus^17^. Taken together it is difficult to conclude on a linear correlation between drebrin expression and seizure susceptibility. Another possibility is that drebrin may be spatially and temporally regulated in response to seizure activity. In these lines, several studies showed changes in drebrin distribution following high-frequency stimulation in vivo, activation of NMDA receptor or induction of LTP with glutamate uncaging^40,46,47^. Interestingly, induction of LTP using glutamate uncaging, led to an initial decrease in drebrin concentration in the dendritic spine when the spine head volume increases, then later drebrin reenters the spine during the phase of actin stabilization^40^. This highly dynamic distribution of drebrin during synaptic activity suggests that drebrin may also change its localization to regulate actin dynamics in the dendritic spine in response to seizure activity to help protect the synapse from dysfunction.

In conclusion, our data demonstrate, in line with previous results on the same drebrin KO mice^13^, that loss of the dendritic spine enriched actin-binding protein drebrin does not alter physiological oscillatory activity. On the contrary, we found that drebrin loss increases the susceptibility of the neuronal network to develop epileptiform discharges. The results confirm that stable actin filaments might have a protecting effect against seizures.

## Experimental procedures

### Animals and slice preparation

DBN KO mice were generated as previously described^13^. Ten Drebrin-KO and 10 wildtype B6/N mice of both sexes were used at an age of 8–12 weeks in the study. Animal procedures were conducted in accordance with the guidelines of the European Communities Council approved by the Berlin Animal Ethics Committee (Landesamt für Gesundheit und Soziales Berlin, T0347/11). The mice were anesthetized with isoflurane and then decapitated. Their brains were removed from the skull and immersed in ice-cold carbogenated (95% O_2_/5% CO_2_) sucrose-based solution with an osmolarity of ~ 330 mosmol/kg containing (in mM): NaCl, 80; NaHCO_3,_ 25; NaH_2_PO_4,_ 1.25; KCl, 2.5; glucose, 25; sucrose, 85; CaCl_2,_ 0.5; MgCl, 3. The brain was cut into 400 μm thick horizontal slices containing both the hippocampus and the entorhinal cortex at an angle of about 13° in the fronto-occipital direction with a vibratome (DSK microslicer DTK-1000, Dosaka, Japan). Slices were immediately transferred to an interface-type recording chamber perfused with carbogenated, warm (36 ± 0.2 °C) artificial cerebrospinal fluid (ACSF; flow rate of 1.7 ± 0.1 ml/min; osmolarity of ~ 300 mosmol/kg) of the following composition (in mM): NaCl, 129; NaH_2_PO_4_, 1.25; NaHCO_3_, 21; glucose, 10; MgSO_4_, 1.8; CaCl_2_, 1.6; KCl, 3. Slices were incubated for at least one hour before starting recordings.

### Electrophysiology

Local field potentials (LFPs) were recorded with glass pipettes filled with ACSF in the stratum pyramidale in CA3b of the hippocampus for recordings of gamma oscillations and in layers IV-V of the medial entorhinal cortex for seizure investigation. Recordings were amplified by an EXT-02 B amplifier (npi, Tamm, Germany), low-pass filtered at 1 kHz and sampled by a CED 1401 interface (Cambridge Electronic Design, Cambridge, UK) at 5 kHz. Gamma oscillations were recorded in AC whereas seizure investigation was done in DC recording mode. For the induction of gamma oscillations in hippocampal slices, kainate (KA, 500 nM, Tocris Bioscience, Bristol, UK) was administered by bath application for 200 min^48^. To induce epileptiform activity in medial entorhinal cortex slices, Mg^2+^ was omitted from the bath solution until the end of the recording (190–200 min)^23,24^.

### Western blot

Following the electrophysiological recordings, slices were harvested, snap frozen and stored at -80° until needed. Each slice was lysed in 100 µl Ripa Buffer (50 mM Tris–HCL pH 7.4, 150 mM NaCl, 0.5% sodium deoxycholate, 1% NP40, 0.1% SDS) supplemented with protease inhibitors (Calbiochem set III) and phosphatase inhibitors (1 mM Na_2_MO_4_,1 mM NaF, 20 mM β-glycerophosphate, 1 mM Na_3_V0_4_, 500 nM Cantharidin) using a tissue homogenizer (Minilys, Bertin instruments). Homogenates were centrifuged at 20,000 G and supernatant was collected for further protein quantification analysis using BCA Thermo Scientific Pierce Protein Assay. 30 µg of protein was loaded on an SDS-PAGE gel and western blot analysis was performed as previously described^49^. Anti-Drebrin (M2F6, Enzo) was used at a concentration of 1:1000 and anti-α-Tubulin (Sigma) was used in 1:8000. Quantification of band densities was performed using FIJI. The area of the band and the mean grey value were measured to obtain a relative density. For relative quantifications, measurements were normalized to tubulin loading control.

### Data evaluation and statistics

This study was conducted in accordance with the ARRIVE guidelines^50^. Gamma oscillations were analyzed by calculating power spectra with a 120-s window every 2 min during the whole recording. Spontaneous activity was computed as the peak power between 20 and 50 Hz during a period of 10 min before induction of the oscillations with KA. In some recordings, spontaneous oscillations were observed during this pre-induction period. Network activity was considered an oscillation when the power spectrum had a peak between 30 and 80 Hz and the Q (quality) factor (frequency/half bandwidth) of the oscillation was higher than the subcritical 0.5^51^. The Q factor measures the periodicity of oscillations independently of the peak frequency (where a high Q factor indicates a sharply distributed oscillation in the power spectrum around the peak frequency and a more periodic, predictable and less dampened oscillation)^52^. KA-induced gamma oscillations were analyzed 20–30 min after induction. Peak power, peak frequency, half bandwidth (at 50% of peak power), and Q factor of the oscillations were extracted by using a custom-made script for the Spike2 software (Cambridge Electronic Design, Cambridge, UK)^51,53^. D'Agostino-Pearson normality test was used to test the Gaussian distribution of the data. Peak power was found to be distributed lognormally, therefore is represented as geometric mean and geometric SD factor^54^. All other normally distributed parameters are presented as (arithmetic) mean ± SEM. The calculated parameters in the WT and KO group were compared with the Student’s t-test. The lognormal distributed power values were first transformed to the logarithms and the logs were analyzed statistically.

Epileptiform activity was analyzed by calculating the following parameters. Seizure onset latency was calculated as the time until the first seizure-like event (SLE) after omitting Mg^2+^ from the extracellular solution^55^. The time until the first appearance of late recurrent discharges (LRD) after zero Mg^2+^ application was used as onset latency of LRD. After appearance, SLEs were analyzed for their incidence (events/min) and duration^56,57^. Statistical comparison of epileptiform activity parameters was done by Student’s t-test. Fisher's exact test was used to compare the fractions of slices developing gamma oscillations or seizure-like activities. Significance level was set at p < 0.05.

## Supplementary Information

Supplementary Information

## Data Availability

The data that support the findings of this study are contained within the article. Not shown data are available on request from the corresponding authors ZG and PK.
