# Supplementary material for: The actin binding protein drebrin helps to protect against the development of seizure-like events in the entorhinal cortex
Source: Sci Rep. 2021 Apr 21;11:8662. doi: 10.1038/s41598-021-87967-5 (PMC8060314; doi:10.1038/s41598-021-87967-5)
Supplement: Supplementary file 1 — Supplementary Information [file 41598_2021_87967_MOESM1_ESM.pdf]

The actin binding protein drebrin helps to protect against the development of seizure-like events in the entorhinal cortex

Alexander Klemz, Patricia Kreis, Britta J. Eickholt, Zoltan Gerevich

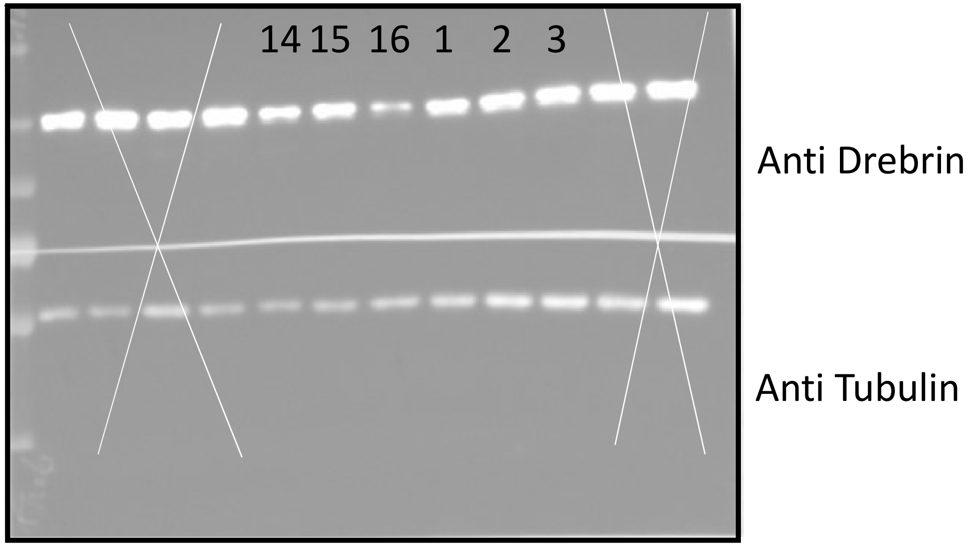

Longer exposure

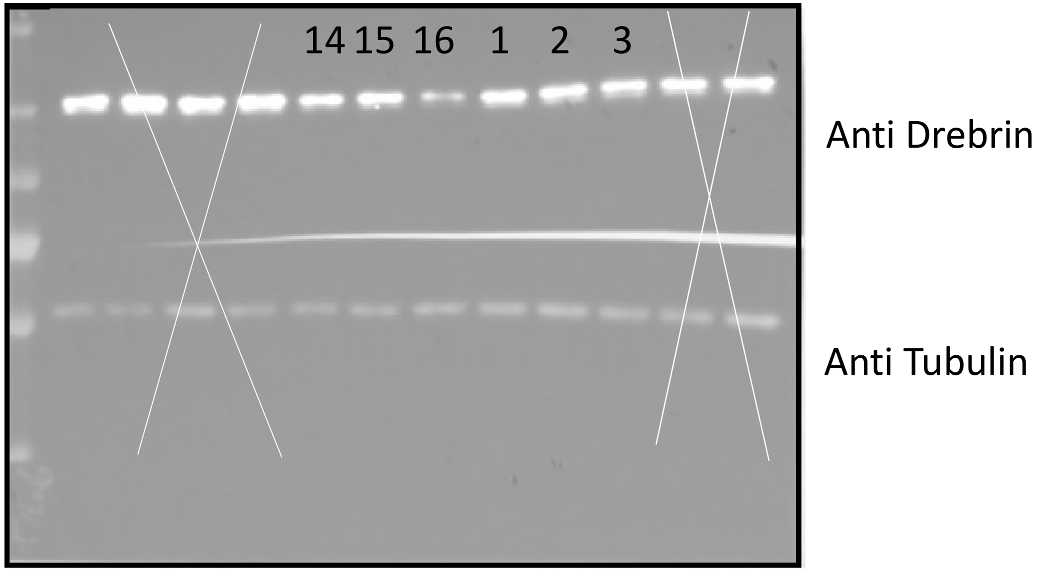

Shorter exposure

Supplementary figure 1. Uncropped western blots related to figure 1g

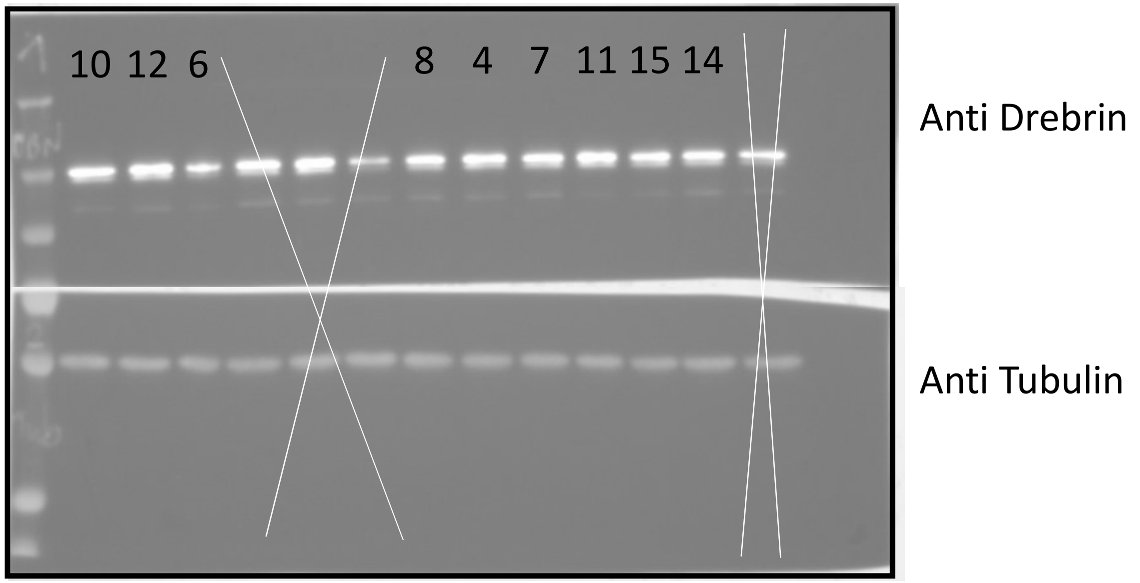

Supplementary figure 2. Uncropped western blots related to figure 3a
